# Red Blood Cell Morphologic Abnormalities in Patients Hospitalized for COVID-19

**DOI:** 10.3389/fphys.2022.932013

**Published:** 2022-07-04

**Authors:** Giacomo Marchi, Claudia Bozzini, Lorenzo Bertolone, Francesco Dima, Fabiana Busti, Annalisa Castagna, Chiara Stranieri, Anna Maria Fratta Pasini, Simonetta Friso, Giuseppe Lippi, Domenico Girelli, Alice Vianello

**Affiliations:** ^1^ Section of Internal Medicine, Department of Medicine, University of Verona, Verona, Italy; ^2^ EuroBloodNet Referral Center for Rare Hematological Disorders, University of Verona, Verona, Italy; ^3^ Section of Clinical Biochemistry, University of Verona, Verona, Italy

**Keywords:** red blood cells (RBCs), erythrocyte membrane, peripheral blood smear, COVID-19, RBC morphology

## Abstract

Peripheral blood smear is a simple laboratory tool, which remains of invaluable help for diagnosing primary and secondary abnormalities of blood cells despite advances in automated and molecular techniques. Red blood cells (RBCs) abnormalities are known to occur in many viral infections, typically in the form of mild normo-microcytic anemia. While several hematological alterations at automated complete blood count (including neutrophilia, lymphopenia, and increased red cell distribution width—RDW) have been consistently associated with severity of COVID-19, there is scarce information on RBCs morphological abnormalities, mainly as case-reports or small series of patients, which are hardly comparable due to heterogeneity in sampling times and definition of illness severity. We report here a systematic evaluation of RBCs morphology at peripheral blood smear in COVID-19 patients within the first 72 h from hospital admission. One hundred and fifteen patients were included, with detailed collection of other clinical variables and follow-up. A certain degree of abnormalities in RBCs morphology was observed in 75 (65%) patients. Heterogenous alterations were noted, with spiculated cells being the more frequent morphology. The group with >10% RBCs abnormalities had more consistent lymphopenia and thrombocytopenia compared to those without abnormalities or <10% RBCs abnormalities (*p* < 0.018, and *p* < 0.021, respectively), thus underpinning a possible association with an overall more sustained immune-inflammatory “stress” hematopoiesis. Follow-up analysis showed a different mortality rate across groups, with the highest rate in those with more frequent RBCs morphological alterations compared to those with <10% or no abnormalities (41.9%, vs. 20.5%, vs. 12.5%, respectively, *p* = 0.012). Despite the inherent limitations of such simple association, our results point out towards further studies on erythropoiesis alterations in the pathophysiology of COVID-19.

## Introduction

Peripheral blood smear is a simple and widely available laboratory tool. Due to the advances in hematology automation, the actual use of peripheral blood smear has steadily decreased in the last decades in parallel with the number of skilled physicians who routinely review the smears. Nevertheless, it remains of invaluable help for diagnosis of primary and secondary alterations of blood cells (Bain 2005). Currently, peripheral blood smear examination is prompted by the clinical suspicion of certain conditions (e.g., hemolysis during thrombotic microangiopathies, or leukemia) or is independently conducted by the laboratory staff as a response to “flags” from the automated complete blood count (CBC) (for example thrombocytopenia or blasts) (for a comprehensive review see Bain 2005).

The COVID-19 pandemic has boosted an unprecedented expansion of research, especially focused on the complex immunological response to severe respiratory syndrome coronavirus 2 (SARS-CoV-2) (Giamarellos-Bourboulis et al., 2020; Brodin 2021). A number of clinical features have been associated with mortality in patients with SARS-CoV-2 infection, including older age, male sex, and obesity (Izcovich et al., 2020). Among CBC parameters, increased white blood cell (WBC) count, thrombocytopenia, lymphopenia, neutrophilia, and eosinopenia have been identified as severity markers (Izcovich et al., 2020; Sun et al., 2020). Sex-, age- and obesity-related immunological alterations, such as low type I interferon (IFN) response, increased inflammasome activity, and elevated production of certain cytokines [IL (interleukin)-12, IL-6, TNF (tumor necrosis factor), IL-1ß], have been hypothesized to play a role in influencing disease severity (Giamarellos-Bourboulis et al., 2020). While WBC appear as obvious pivotal pathogenic drivers of disease progression, scarce information is available on red blood cells (RBCs) abnormalities in COVID-19. Systemic inflammation profoundly affects erythropoiesis and iron metabolism, especially through TNF-mediated erythroid progenitor suppression (Ganz 2019) and IL-6-mediated hepcidin upregulation, thus resulting in macrophage iron retention and hypoferremia (Girelli et al., 2021). Anemia is commonplace in COVID-19, but conflicting data are available about its correlation with disease severity (Wang et al., 2020; Zhou et al., 2020). Interestingly, elevated RBCs distribution width (RDW), which may reflect erythropoietic stress, has been more clearly associated with poor prognosis (Bergamaschi et al., 2021; Lippi et al., 2021; Sarkar et al., 2022).

Regarding blood cells morphology, relatively few data are available so far, most of them primary focusing on WBC morphology. Acquired Pelger-Huët anomaly, monolobate neutrophils, atypical lymphocytes with plasmacytoid features (Nazarullah et al., 2020), leukoerythroblastosis (Mitra et al., 2020), large monocytes with vacuolated cytoplasm (Zhang et al., 2021), macrothrombocytes (Rampotas and Pavord, 2021) and microthrombi (Ackermann et al., 2020) have been described, mostly in severe COVID-19 cases. As regards to RBCs morphology, a number of abnormalities have been described, including polichromasia, basophilic stippling, rouleaux, auto-agglutination, spherocytes, schistocytes, stomatocytes, knizocytes, and mushroom-shaped cells (Berzuini et al., 2021; Gerard et al., 2021), usually without association with hemolysis. All these studies were case reports (Khakwani et al., 2021) or examined a limited number of peripheral blood smears of COVID-19 patients with different disease severity and at different time points. For example, Berzuini et al. (2021) studied 20 COVID-19 patients with mild to severe anemia (Hb 72–105 g/L) at the time of referral for pre-transfusion testing, with long duration of hospital stay (median 30 days, range 18–50 days). Starting from an intriguing observation of mushroom-shaped cells, Gerard et al. (2021) found frequent abnormalities in 50 COVID-19 patients (the majority recruited from Intensive Care Unit – ICU), but description of other findings was scanty. Overall, such studies are hardly comparable, and little or no information was available regarding the other clinical and laboratory features of the patients investigated.

Here we systematically evaluated RBCs morphology abnormalities in peripheral blood smear of COVID-19 patients at hospital admission. We also collected clinical features and in-hospital outcomes in order to explore potential associations between blood smear patterns and disease course.

## Materials and Methods

We obtained the peripheral blood smear of 116 patients aged 18 years or older affected by COVID-19 and hospitalized in COVID Units at the Verona University Hospital between April and December 2020. All the samples were collected within the first 72 h of hospital admission, processed by an analyzer connected to equipment (SP-10 for slide creation and staining and the DI-60 automated digital cell imaging analyzer; Sysmex Corporation, Kobe, Japan) to automatically generate a May-Grünwald Giemsa-stained peripheral blood smear and capture high-magnification images in the optimal area for RBC (i.e., where RBC are more or less evenly spaced and central pallor is appreciated). The peripheral blood smears were then stored at room temperature. Images were acquired with an image analysis and, in case of non-optimal automatic preparation, with light optical microscope. One patient has been excluded from the analysis since he was discharged from the Verona University Hospital within a week after admission and transferred to a secondary hospital for acute patients for further treatment with unknown final in-hospital outcome. Demographic characteristics, past medical history, biochemical parameters, and arterial blood gas test were collected at hospital admission. Information about in-hospital evolution were retrospectively collected from the medical records.

**FIGURE 1:**
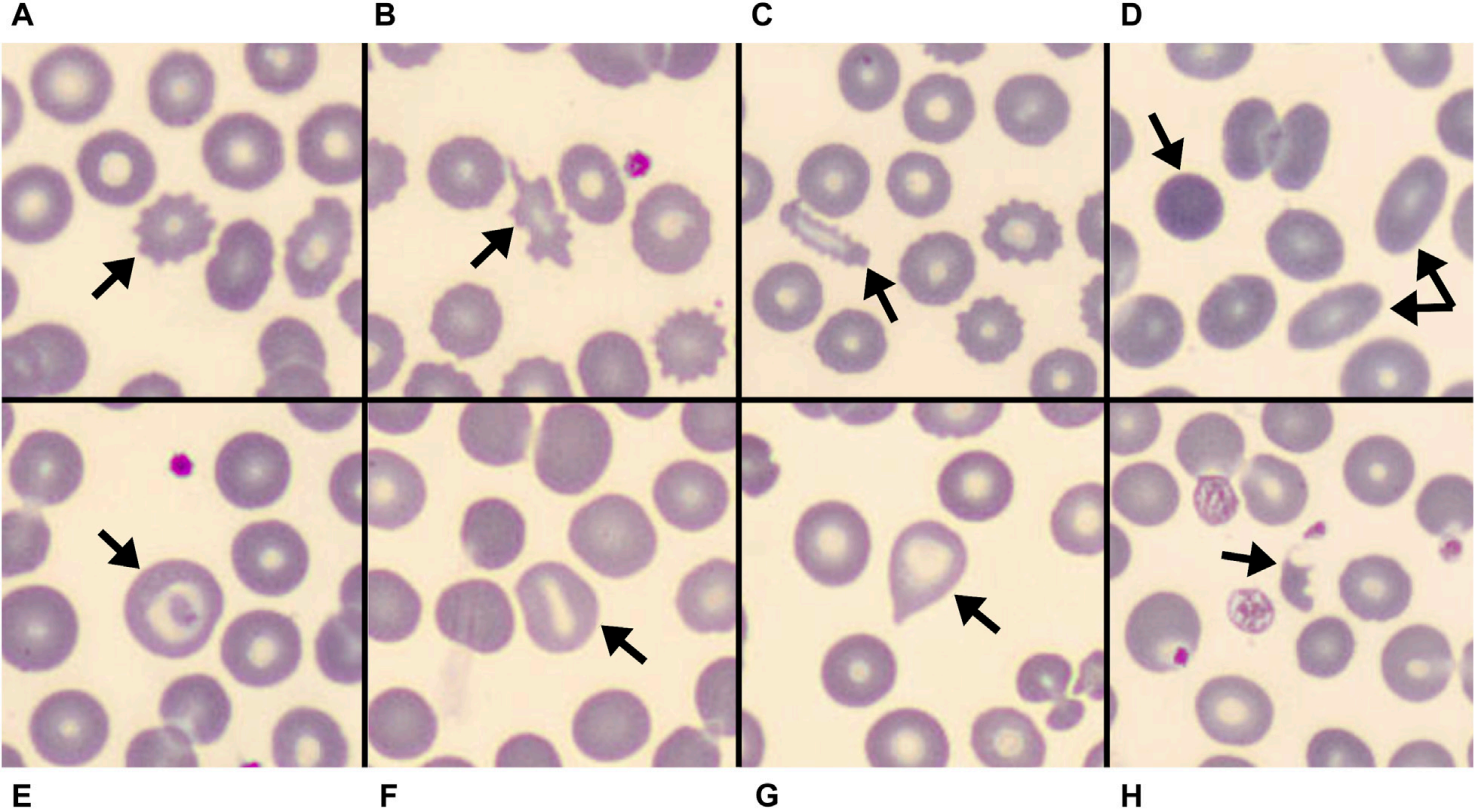
Some of the erythrocyte’s shapes observed at peripheral blood smear of COVID-19 patients at hospital admission are shown. **(A–C)** spiculated cells: **(A)**: echinocyte; **(B)**: acanthocyte; **(C)**: spiculated elliptocyte. **(D)**: spherocyte and two elliptocytes. **(E)**: target cell. **(F)**: stomatocyte. **(G)**: dacriocyte. **(H)**: schistocyte.

**FIGURE 2:**
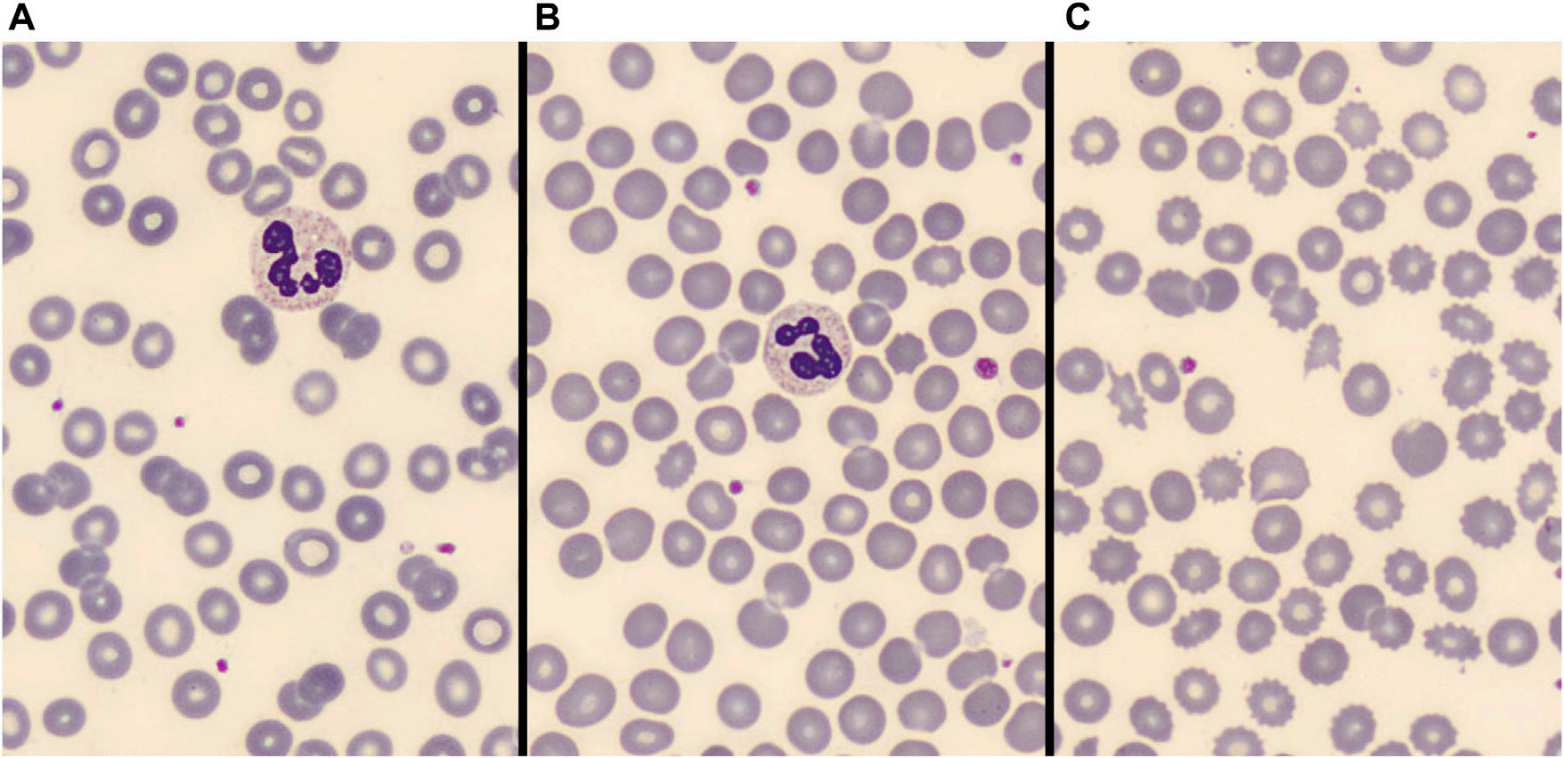
**(A)**: no significative abnormal RBC morphologies. **(B)**: <10% abnormal RBC morphologies. **(C)**: >10% abnormal RBC morphologies.

Two independent skilled operators reviewed the blood smear images. RBCs morphology analysis was firstly performed by GM, a physician skilled in peripheral blood smear examination. To ensure a sufficient stability of subgroup’s classification, about 3,000 RBCs were examined in each blood smear. The number of RBCs morphological alterations (including acanthocytes, echinocytes, spherocytes, elliptocytes, schistocytes, stomatocytes, target cells, and dacriocytes; see Figure 1 for some examples) was annotated. At least some abnormal morphologies were detected in near two thirds (75 out of 115) patients. Therefore, for subsequent statistical analysis patients were categorized into three groups according to the magnitude of abnormal RBC morphologies: no alterations, alterations in ≤10% of RBCs, and alterations involving >10% of RBCs as shown in Figure 2. Results were cross validated by a second physician (CB), who independently reviewed the peripheral blood smears.

The study protocol, as part of a wider ongoing study on iron metabolism in COVID-19, was approved by the Ethical Committee of the University Hospital of Verona (No. 2646CESC). Due to the exceptional pandemic circumstances, the Ethical Committee waived the requirement of written informed consent for participation in observational and non-interventional studies. Oral informed consent with annotation in the medical records was considered sufficient.

### Statistical Analysis

Continuous variables are presented as mean ± standard deviation (SD) if normally distributed or as median (interquartile range) if the Shapiro-Wilk test determined the not-normal distribution of the variables. Categorical variables are presented as frequencies (percentages).

Variables were tabulated according to study groups. Intergroup differences in normally distributed quantitative variables were assessed with analysis of variance (one-way ANOVA). Not-normal quantitative variables were compared with the Kruskall-Wallis test. Categorical data were compared with the Chi-square test or Fisher’s exact test, as appropriate.

A two-sided *p* < 0.05 was considered statistically significant. All statistical analyses were performed using IBM SPSS 28.0 software (SPSS Inc., Chicago, IL, United States).

## Results

One hundred and fifteen patients hospitalized for COVID-19 (90.4%, *n* = 104 with clinical features and imaging consistent with pneumonia) were included in this analysis. Table 1 describes the patient characteristics at baseline. Most of them had at least one comorbidity (87.8%), chronic kidney disease (CKD) or chronic liver disease (CLD) being present in 15 (13.0%) and 4 (3.5%) patients, respectively. In six subjects (5.2%) an active hematologic neoplasia was reported (one Waldenstrom macroglobulinemia, one non-Hodgkin B cell lymphoma, one multiple myeloma IgG kappa, one splenic marginal zone lymphoma with 10%–20% of bone marrow infiltration, one MALT lymphoma with concomitant CKD, one lymphocytic lymphoma with associated CLD). Three patients (2.6%) had a history of chronic anemia (one beta-thalassemia, one iron deficiency anemia, one multifactorial anemia associated with CKD). Supplementary Table S1 reports a detailed list of all the comorbidities in the entire cohort and in different study groups.

**TABLE 1:** Baseline demographic, clinical and biochemical characteristics of the entire cohort and according to the presence of RBCs abnormalities at the peripheral blood smear.

	Entire cohort	Abnormal RBC morphologies			
		No (*n* = 40)	<10% (*n* = 44)	>10% (*n* = 31)	*p*-value
Demographic and clinical characteristics					
Age (years)	75 [62–83]	71 [62–82]	78 [59–83]	79 [65–87]	0.292
Gender (Male)	72 (62.6)	23 (57.5)	28 (63.6)	21 (67.7)	0.665
Comorbidities (any)	101 (87.8)	36 (90.0)	41 (93.2)	24 (77.4)	0.122
Chronic kidney disease	15 (13.0)	3 (7.5)	4 (9.1)	8 (25.8)	0.064
Chronic liver disease	4 (3.5)	1 (2.5)	1 (2.3)	2 (6.5)	0.676
Active hematologic neoplasia	6 (5.2)	2 (5.0)	1 (2.3)	3 (9.7)	0.382
Chronic anemia	3 (2.6)	1 (2.5)	0	2 (6.5)	0.187
Time from symptoms onset to hospital admission (days)	6 [3–9]	6 [3–8]	7 [5–10]	5 [2–9]	0.367
Peripheral O_2_ Saturation (%)	93 [90–96]	92 [90–95]	94 [91–96]	91 [88–94]	**0.046**
Heart rate (bpm)	89 (±18.1)	90 (±15.1)	88 (±17.2)	88 (±23.0)	0.827
Systolic blood pressure (mmHg)	135 (±20.9)	137 (±19.0)	137 (±21.6)	129 (±21.6)	0.198
Temperature (°C)	37.4 (±1.0)	37.4 (±0.9)	37.3 (±1.2)	37.6 (±1.0)	0.544
Biochemical characteristics					
Hemoglobin (g/L)	128 [119–138]	131 [110–141]	129 [123–137]	126 [113–138]	0.707
MCV (fL)	89.2 [87.0–92.9]	90.0 [87.3–94.2]	89.2 [86.8–92.7]	89.0 [86.7–93.6]	0.659
RDW (%)	13.8 [12.9–14.9]	13.6 [12.6–14.7]	13.6 [12.9–14.6]	14.6 [13.4–15.4]	0.173
White Blood Cells (10^9^/L)	7.68 [4.71–11.38]	7.12 [5.00–11.78]	8.15 [5.11–11.37]	6.66 [4.36–12.14]	0.848
Neutrophils (10^9^/L)	6.13 [3.50–9.83]	5.63 [3.42–9.74]	6.79 [3.66–10.05]	5.73 [3.47–9.58]	0.814
Lymphocytes (10^9^/L)	0.77 [0.51–1.06]	0.81 [0.59–1.18]	0.80 [0.48–1.13]	0.59 [0.35–0.86]	**0.018**
Platelets (10^9^/L)	206 [157–275]	212 [157–302]	234 [168–282]	179 [148–213]	**0.021**
C reactive protein (mg/L)	75 [37–147]	75 [34–144]	67 [33–187]	112 [48–178]	0.245
Ferritin (µg/L)	664 [350–1,139]	545 [358–1,250]	639 [273–928]	806 [397–1,247]	0.374
Iron (µg/dl)	38 [23–60]	38 [23–66]	38 [22–63]	37 [24–52]	0.965
Transferrin (g/L)	1.52 [1.26–1.90]	1.58 [1.34–1.95]	1.54 [1.27–1.88]	1.40 [1.18–1.91]	0.349
Transferrin saturation (%)	17 [11–29]	16 [10–28]	18 [11–30]	19 [12–30]	0.680
Fibrinogen (g/L)	5.59 [4.61–7.22]	5.46 [4.77–7.11]	5.55 [4.24–7.02]	6.09 [4.61–7.52]	0.749
Creatinine (mg/dl)	0.85 [0.71–1.13]	0.82 [0.70–1.09]	0.88 [0.77–1.20]	0.89 [0.70–1.39]	0.466
eGFR, (mL/min/1.73 m^2^)	81 [54–92]	82 [59–94]	80 [55–91]	76 [44–94]	0.499
ALT (U/L)	29 [19–46]	30 [19–47]	33 [21–43]	26 [15–46]	0.569
Total bilirubin (mg/dl)	0.53 [0.36–0.72]	0.40 [0.32–0.64]	0.56 [0.38–0.77]	0.56 [0.48–0.80]	0.110
Albumin (g/L)	35.3 (±4.6)	36.6 (±3.5)	35.5 (±4.5)	33.5 (±5.4)	**0.036**
Arterial blood gases at ED presentation					
PaO_2_/FiO_2_ (ratio)	262 [229–314]	276 [238–324]	262 [238–314]	233 [196–318]	0.282
Lactate (mmol/L)	1.3 [0.9–1.9]	1.2 [1.1–2.2]	1.2 [1.0–1.7]	1.4 [0.8–2.3]	0.771

Data are presented as n (%), mean (±SD) or median [interquartile range]. Statistical significance was set at *p* < 0.05. *p* values < 0.05 are indicated in bold.

MCV, mean corpuscular volume; RDW, red blood cell distribution width; eGFR, estimated glomerular filtration rate (CKD, formula); ALT, alanin aminotransferase; ED, Emergency department; PaO2, arterial partial oxygen tension; FiO2, inspiratory oxygen fraction.

Patients presented at Emergency Department (ED) with median peripheral O_2_ Saturation (SpO_2_) of 93% [90–96], mean heart rate of 89 bpm (±18.1), mean systolic blood pressure of 135 mmHg (±20.9) and mild temperature elevation [37.4°C (±1.0)]. The median time from COVID-19 symptoms onset to hospital admission was 6 days [3–9]. Except for a significantly different distribution of SpO_2_, there were no other statistically significative differences between groups for demographic and clinical characteristics at hospital presentation. Patients in the group with more RBCs morphological abnormalities had lowest peripheral O_2_ saturation at hospital admission (*p* = 0.046; Table 1), and all required supplementary O_2_ in different modalities during hospitalization (Table 2).

**TABLE 2:** In-hospital evolution of the entire cohort and according to the presence of abnormal RBC morphologies.

	Entire cohort	Abnormal RBC morphologies			
		No (*n* = 40)	<10% (*n* = 44)	>10% (*n* = 31)	*p*-value
Evolution					
Length of hospitalization (days)	13 [9–21]	10 [8–18]	14 [10–28]	14 [9–22]	0.123
ARDS	5 (4.3)	1 (2.5)	1 (2.3)	3 (9.7)	0.309
Need for RBCs transfusion	12 (10.4)	3 (7.5)	4 (9.1)	5 (16.1)	0.483
Maximal O_2_ therapy needed					
No O_2_ therapy needed	13 (11.3)	5 (12.5)	8 (18.2)	0	
Standard O_2_ therapy^a^	71 (61.7)	29 (72.5)	22 (50.0)	20 (64.5)	
Ventilation/Intubation^b^	31 (27.0)	6 (15.0)	14 (31.8)	11 (35.5)	
ICU admission	16 (13.9)	1 (2.5)	9 (20.5)	6 (19.4)	**0.021**
In-hospital death	27 (23.5)	5 (12.5)	9 (20.5)	13 (41.9)	**0.012**

Data are presented as n (%) or median [interquartile range]. Statistical significance was set at *p* < 0.05. *p* values < 0.05 are indicated in bold.

ARDS, acute respiratory distress syndrome; RBCs, red blood cells; ICU, intensive care unit.

a Oxygen delivered through nasal cannula, Venturi mask or reservoir mask.

b Non-invasive ventilatory support or orotracheal intubation.

An arterial blood gas sample was collected at ED for 85 patients. Median PaO_2_/FiO_2_ ratio for the entire cohort was 262 [229–314]. Although a trend toward reduction of the PaO_2_/FiO_2_ ratio was observed with increasing RBCs morphological abnormalities, the differences did not meet statistical significance (Table 1). Among the biochemical parameters, most did not differ significantly across groups (Table 1), with some noticeable exceptions represented by lymphocytes, platelets, and albumin. In particular, the subset of patients showing alterations in >10% of RBCs had significantly lower median lymphocyte [0.59 × 10^9^/L (0.35–0.86), *p* = 0.018] and platelet [179 × 10^9^/L (148–213), *p* = 0.021] counts, as well as lower mean albumin levels [33.5 g/L (±5.4), *p* = 0.036] compared to the other groups. Hemoglobin (Hb) levels and RDW did not differ significantly among groups, while there was a trend towards lower (Hb) and higher (RDW) levels in the group with >10% RBCs alterations. The same was observed for other biochemical parameters (e.g., C reactive protein, ferritin, fibrinogen), likely reflecting a tendency toward a more severe COVID-19 disease in those with more RBCs abnormalities at presentation. A graphical summary of the trend of most relevant biochemical parameters stratified according to RBCs morphological alterations subgroups are shown in Supplementary Figures S1, S2.

Peripheral blood smears observation revealed almost all the previously described abnormal RBC morphologies (Figure 1). Schistocytes, stomatocytes, target cells, and dacriocytes were uncommon and usually sporadic when detected. Indirect signs of RBCs membrane abnormalities were also frequently seen, like rouleaux. Spiculated cells were the most common and typical finding, showing borderline features between acanthocytes and echinocytes. These were often associated with other abnormal morphologies, even concurrently observed in the single RBC (e.g., spiculated elliptocytes as in Figure 1C).

Alterations of WBC and platelets were also seen, like neutrophil and monocyte abnormalities and large/giant PLTs and PLT aggregates, but their categorization appeared less reproducible and out of the scope of this manuscript. The division into the three groups according to the degree of abnormal RBC morphologies mainly reflects the presence and the degree of spiculated cells.

Table 2 reports the most relevant clinical outcomes during in-hospital stay stratified as per the RBCs morphological alteration groups. Median length of hospitalization did not significantly differ among groups, although patients with no RBCs alterations tended to experience a median shorter hospitalization compared to the other groups. A significant association was observed between peripheral blood smear groups and relevant clinical outcomes, such as ICU admission, and in-hospital death. In particular, the lowest proportion of in-hospital deaths (12.5%, *n* = 5; *p* = 0.012 compared to the other groups) was observed in patients with no RBCs morphological alterations on basal blood smear, and only one patient in this group required ICU admission (2.5%). The highest in-hospital mortality rate was observed in the group of patients with >10% RBCs morphological abnormalities (41.9%, *n* = 13; *p* = 0.012).

No difference was observed among study groups in the proportion of patients admitted to ICU or dead during the in-hospital stay (Supplementary Table S2). As expected, comorbidities were more frequent in non-survivors. In particular, in the group with no RBCs abnormalities one patient had chronic liver disease and another had an active hematologic neoplasia. Two patients showing <10% morphologic alterations in RBCs had chronic kidney disease, while one patient of the same group had chronic liver disease. Of the six comorbid patients of the group with >10% of RBCs abnormalities, four had chronic kidney disease, one reported chronic anemia and one was affected by both chronic kidney disease and active hematologic neoplasia.

## Discussion

Peripheral blood smear represents a simple and widely available tool, frequently overlooked in the current era of automated analyses. A few previous reports consistently described RBCs morphologic alterations in hospitalized COVID-19 patients, but results were scanty and hardly comparable. We confirm a high frequency of RBCs morphologic abnormalities in COVID-19 patients. Of note, at variance with previous reports which mainly focused on patients with longstanding severe disease and/or anemia requiring blood transfusions, we obtained peripheral blood smears during the early phase of hospitalization and could collect detailed data regarding the disease’s course. We noticed an association between the presence of RBCs alterations, worse disease presentation (significative lower SpO_2_, albumin and lymphocyte and platelet counts) and outcomes (significative higher ICU admission and in-hospital deaths). While no causal inference is possible, this association represents an intriguing finding that may deserve further exploration. No clear association was found between the degree of RBCs morphological abnormalities and median Hb or RDW levels, although the group with >10% of RBCs abnormalities displayed a trend toward lower Hb and higher RDW.

This study has several limitations, including the arbitrary cut-off for categorization and single-center review of the smears, which may be not fully reproducible. Unfortunately, we had no chance to collect a further smear during hospitalization. Small group sizes did not allow an appropriate adjustment for the single comorbidities, including those potentially leading per se to RBCs abnormalities, i.e., chronic kidney or liver disease, active hematologic neoplasia or chronic anemia. Other limits are the lack of a control group of non-COVID patients with respiratory infections and a not certain applicability of our findings to later SARS-CoV-2 variants and to vaccinated patients, as the observation period corresponds to first and second waves of Italian SARS-CoV-2 pandemic.

On the other hand, to the best of our knowledge, this is the largest study reporting RBCs morphology in COVID-19 at hospital presentation.

The most typical finding was the presence of spiculated RBCs, with overlapping features between echinocytes and acanthocytes. Similar cells have been described in COVID-19 also by others (Khakwani et al., 2021). Spiculated cells are known to originate from changes in the organization of RBC membrane components such as defects in the lipid and/or protein composition (Mentzer, 2022). Apparently in contrast with our observations, Berzuini et al. (2021) reported stomatocytes and knizocytes as the most common finding (70%) in their series of COVID-19 patients. However, their observations focused on severely ill patients referred for pre-transfusion test, with longstanding hospital stay and a more pronounced decrease of hemoglobin levels. We may hypothesise that spiculated cells could reflect early changes in the RBC membrane composition induced by SARS-CoV-2 infection, while stomatocytes and knizocytes may reflect a more advanced damage with loss of elastic properties. However, a comparative analysis of RBC morphology and physiology in well clinically characterized groups would be needed to confirm this hypothesis. Interestingly, it has been showed that SARS-CoV-2 is able to invade erythroid precursors (Shahbaz et al., 2021), and that stress erythropoiesis is an inherent component of the hematopoietic response to SARS-CoV-2 infection along with neutrophilia and lymphopenia (recently reviewed in Elahi 2022). Indeed, as shown on Table 1, both lymphocyte and platelet counts were lower in the group with the more pronounced RBCs morphological abnormalities.

As mentioned above, the high prevalence of spiculated RBCs points towards membrane alterations. Deformability defects have been demonstrated in erythrocytes of COVID-19 patients, and they are known to impair oxygen delivery (Kubánková et al., 2021). Of note, recent studies have also demonstrated that ferritin, which is typically elevated and prognostically relevant in severe COVID-19 (Girelli et al., 2021), may be implicated. In particular, the heavy chain (FTH1) has been proposed to modulate cytoskeleton integrity in erythroid precursors, favoring microtubules disruption in iron restriction conditions (Goldfarb et al., 2021).

Several surface markers and functional alterations have been described in blood cells of COVID-19 patients (for a review see Ahmadi et al., 2022). Complement activation products, such as C3b and C4d, and viral antigens have been described on RBCs surface in patients with COVID-19 (Lam et al., 2020). Berzuini et al. (2020) observed also that nearly half of patients with COVID-19 had a positive direct antiglobulin test (DAT), and their eluates did not react with test cells but reacted with erythrocytes of other COVID-19 DAT negative patients.

Noteworthy, a comprehensive proteomic, metabolomic, and lipidomic analysis of RBCs from COVID-19 patients has shown relevant structural membrane abnormalities at the protein and lipid levels in COVID-19 patients (Thomas et al., 2020). In particular, the Authors described increased levels of glycolytic intermediates, significant oxidation and fragmentation of ankyrin, spectrin beta, and the N-terminal cytosolic domain of band 3 (AE1) (Thomas et al., 2020). They also observed significantly altered lipid metabolism, especially of short- and medium-chain saturated fatty acids, acyl-carnitines, and sphingolipids (Thomas et al., 2020). These findings suggest that, beyond the immuno-inflammatory mediated stress erythropoiesis, the high level of oxidative stress in severe COVID-19 (Pasini et al., 2021) may also play a role.

Considering the comorbidities that are expected to have a greater impact on RBCs morphology, i.e., chronic kidney or liver disease, active hematologic neoplasia or chronic anemia, no difference was observed among study groups in the proportion of patients admitted to ICU or deceased during the in-hospital stay (Supplementary Table S2).

Altogether these observations support the hypothesis that abnormal RBC morphologies found in acute COVID-19 patients are associated with the ongoing viral infection, rather than reflecting the effect of previous blood, kidney or liver diseases. Peripheral blood smear should be considered as an adjunctive tool to assess prognosis, even in low-income countries, where availability of automated laboratory prognostic markers may be limited.

## Conclusion

Hospitalized patients with COVID-19 frequently have RBCs morphologic abnormalities at admission, mainly in the form of spiculated cells, which may reflect an impairment of protein and lipid membrane composition of erythrocytes. The degree of abnormalities was associated with severity of disease, making peripheral blood smear a possible additional prognostic tool for COVID-19 patients. Whether and how RBCs alterations may be linked to the pathophysiology of severe COVID-19 deserves further studies.

## Data Availability

The raw data supporting the conclusion of this article will be made available by the authors, without undue reservation.
