# Supplementary material for: Red Blood Cell Morphologic Abnormalities in Patients Hospitalized for COVID-19
Source: Front Physiol. 2022 Jul 4;13:932013. doi: 10.3389/fphys.2022.932013 (PMC9289213; doi:10.3389/fphys.2022.932013)
Supplement: Supplementary file 1 [file Table1.DOCX]

**Supplementary Table 1**. Detailed description of comorbidities for the entire cohort and according to the presence of RBCs abnormalities at the peripheral blood smear.

|  | **Entire cohort** | **Abnormal RBC morphologies** | | |  |
| --- | --- | --- | --- | --- | --- |
|  |  | **No (n=40)** | **< 10% (n=44)** | **> 10% (n=31)** | **p-value** |
| Chronic kidney disease | 15 (13.0) | 3 (7.5) | 4 (9.1) | 8 (25.8) | 0.064 |
| Chronic liver disease | 4 (3.5) | 1 (2.5) | 1 (2.3) | 2 (6.5) | 0.676 |
| Active hematologic neoplasia | 6 (5.2) | 2 (5.0) | 1 (2.3) | 3 (9.7) | 0.382 |
| Chronic anemia | 3 (2.6) | 1 (2.5) | 0 | 2 (6.5) | 0.187 |
| Arterial hypertension | 70 (60.9) | 27 (67.5) | 25 (56.8) | 18 (58.1) | 0.564 |
| Atrial fibrillation | 16 (13.9) | 3 (7.5) | 7 (15.9) | 6 (19.4) | 0.305 |
| Chronic heart disease | 17 (14.8) | 2 (5.0) | 7 (15.9) | 8 (25.8) | **0.048** |
| Diabetes | 29 (25.2) | 9 (22.5) | 13 (29.5) | 7 (22.6) | 0.702 |
| Active solid neoplasia | 5 (4.3) | 1 (2.5) | 1 (2.3) | 3 (9.7) | 0.309 |
| COPD | 5 (4.3) | 2 (5.0) | 2 (4.5) | 1 (3.2) | 1.0 |
| Asthma | 3 (2.6) | 2 (5.0) | 1 (2.3) | 0 | 0.626 |
| Rheumatological diseases | 4 (3.5) | 1 (2.5) | 3 (6.8) | 0 | 0.451 |
| HIV infection | 0 | 0 | 0 | 0 | - |
| Immunodepression (other than HIV) | 1 (0.9) | 0 | 0 | 1 (3.2) | 0.230 |
| Thyroid diseases (hypothyroidism) | 11 (9.6) | 3 (7.5) | 3 (6.8) | 5 (16.1) | 0.412 |
| Cerebrovascular diseases | 4 (3.5) | 2 (5.0) | 1 (2.3) | 1 (3.2) | 0.830 |
| Cognitive decay | 14 (12.2) | 6 (15.0) | 4 (9.1) | 4 (12.9) | 0.721 |
| Psychiatric disorders | 5 (4.3) | 3 (7.5) | 2 (4.5) | 0 | 0.370 |
| Comorbidities (any, at least one) | 101 (87.8) | 36 (90.0) | 41 (93.2) | 24 (77.4) | 0.122 |

Data are presented as n (%). Statistical significance was set at p<0.05.

*Abbreviations*: COPD: chronic obstructive pulmonary disease; HIV: human immunodeficiency virus; RBCs: red blood cells.

Rheumatological diseases included 1 case of psoriatic arthritis, 1 case of rheumatoid arthritis, 1 case of pulmonary sarcoidosis and 1 case of seronegative spondylarthritis.

The immunocompromised patient was affected by hypogammaglobulinemia.

All patients reporting thyroid diseases were affected by hypothyroidism.
