# Supplementary material for: Red Blood Cell Morphologic Abnormalities in Patients Hospitalized for COVID-19
Source: Front Physiol. 2022 Jul 4;13:932013. doi: 10.3389/fphys.2022.932013 (PMC9289213; doi:10.3389/fphys.2022.932013)
Supplement: Supplementary file 2 [file Table2.DOCX]

**Supplementary Table 2**. Distribution of comorbidities that are expected to have a greater impact on RBCs morphology in patients admitted to Intensive Care Unit or deceased during the in-hospital stay. Data are shown for the entire cohort and grouped according to the presence of abnormalities of RBCs membrane.

|  | **Entire cohort** | **Abnormal RBC morphologies** | | |
| --- | --- | --- | --- | --- |
|  |  | **No (n=40)** | **< 10% (n=44)** | **> 10% (n=31)** |
| Patients admitted to ICU | 16 (13.9) | 1 (2.5) | 9 (20.5) | 6 (19.4) |
| Patients admitted to ICU, with at least one comorbidity | 2 (12.5) | 0 | 1^a^ (11.1) | 1^a^ (16.7) |
| Patients deceased during in-hospital stay | 27 (23.5) | 5 (12.5) | 9 (20.5) | 13 (41.9) |
| Patients deceased during in-hospital stay, with at least one comorbidity | 11 (40.7) | 2^b,c^ (40.0) | 3^a,b^ (33.3) | 6^a,c,d^ (46.2) |

Data are presented as n (%).

*Abbreviations*: ICU: Intensive Care Unit.

Comorbidities that are known to potentially have an impact on RBCs morphology were considered, as listed in Table 1. a: chronic kidney disease; b: chronic liver disease; c: active hematologic neoplasia; d: chronic anemia.

The proportion of patients with at least one comorbidity was not statistically different among study groups for both ICU admission and in-hospital mortality.
