# Supplementary material for: Red Blood Cell Morphologic Abnormalities in Patients Hospitalized for COVID-19
Source: Front Physiol. 2022 Jul 4;13:932013. doi: 10.3389/fphys.2022.932013 (PMC9289213; doi:10.3389/fphys.2022.932013)
Supplement: Supplementary file 3 [file Image1.PDF]

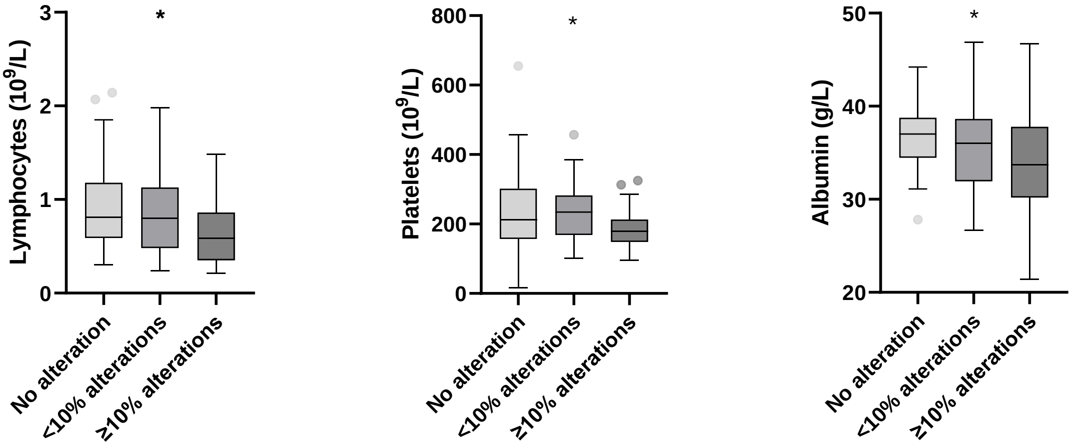

Supplementary Figure 1. Boxplots showing the 25th, 50th and 75th percentiles (box); 10th and 90th percentiles (whiskers); and outliers (circles) of lymphocytes, platelets, and albumin, stratified according to the RBCs morphological abnormalities subgroups. * P-values: 0.018, 0.021, and 0.036, respectively.

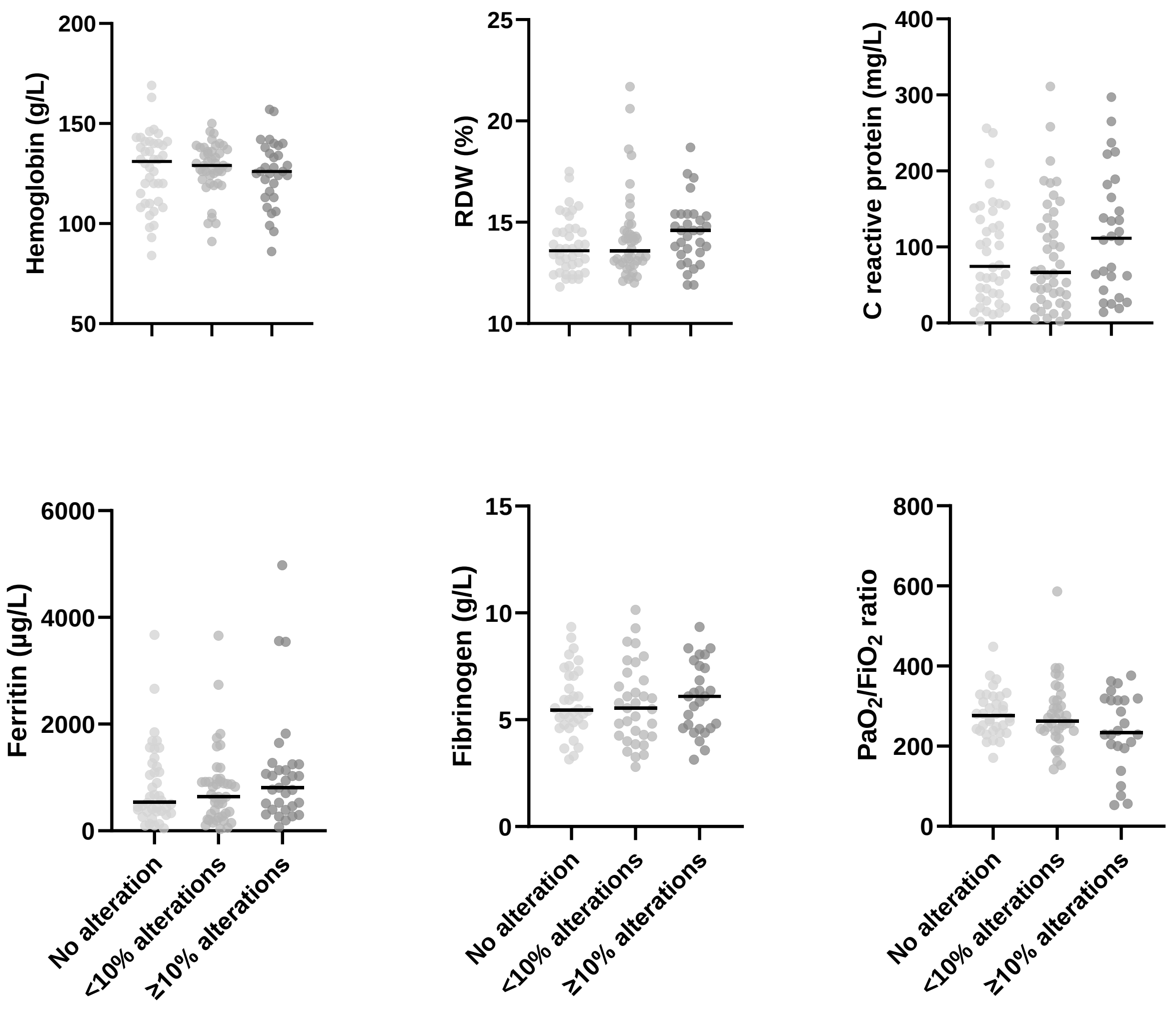

Supplementary Figure 2. Graphical overview of biochemical and arterial blood gas relevant parameters stratified according to the RBCs morphological abnormalities subgroups. Data points (circles) and median (line) of hemoglobin, red blood cells distribution width (RDW), C reactive protein, ferritin, fibrinogen, and arterial partial oxygen tension to inspiratory oxygen fraction ratio (PaO_2/ FiO_2).
